# SARS-CoV-2 Spike protein peptides displayed in the *Pyrococcus furiosus* RAD system preserve epitopes antigenicity, immunogenicity, and virus-neutralizing activity of antibodies

**DOI:** 10.1038/s41598-023-43720-8

**Published:** 2023-10-05

**Authors:** Victor Bolsanelli Cioffi, Maria Fernanda de Castro-Amarante, Aleksei Lulla, Robert Andreata-Santos, Mario Costa Cruz, Ana Carolina Ramos Moreno, Mariângela de Oliveira Silva, Bianca de Miranda Peres, Lucio Holanda Gondim de Freitas Junior, Carolina Borsoi Moraes, Edison Luiz Durigon, Nicola Coker Gordon, Marko Hyvönen, Luís Carlos de Souza Ferreira, Andrea Balan

**Affiliations:** 1 Laboratory of Applied Structural Biology, Department of Microbiology, Institute of Biomedical Sciences, University of São Paulo, Av. Prof. Lineu Prestes, 1374, São Paulo, 05508-000 Brazil; 2 Laboratory of Vaccine Development, Department of Microbiology, University of São Paulo, Institute of Biomedical Sciences, Av. Prof. Lineu Prestes, 1374, São Paulo, 05508-000 Brazil; 3 Department of Biochemistry, University of Cambridge, 80 Tennis Court Road, Cambridge, CB2 1GA UK; 4 Core Facilities to Support Research (CEFAP), Institute of Biomedical Sciences, University of São Paulo, Av. Prof. Lineu Prestes, São Paulo, 173005508-000 Brazil; 5 Vaccine Development Laboratory, Butantan Institute, Av. Vital Brasil, 1500, São Paulo, SP 05503-900 Brazil; 6 Phenotypic Screening Platform, Department of Microbiology, University of São Paulo, Institute of Biomedical Sciences, Av. Prof. Lineu Prestes, 1374, São Paulo, 05508-000 Brazil; 7 Laboratory of Clinical and Molecular Virology, Institute of Biomedical Sciences, University of São Paulo, Av. Prof. Lineu Prestes, 1374, São Paulo, 05508-000 Brazil; 8 Institut Pasteur de São Paulo, Av. Prof. Lucio Martins Rodrigues, 370, São Paulo, 05508-020 Brazil

**Keywords:** Biophysical methods, Infectious-disease diagnostics, Virology

## Abstract

Amongst the potential contribution of protein or peptide-display systems to study epitopes with relevant immunological features, the RAD display system stands out as a highly stable scaffold protein that allows the presentation of constrained target peptides. Here, we employed the RAD display system to present peptides derived from the SARS-CoV-2 Spike (S) protein as a tool to detect specific serum antibodies and to generate polyclonal antibodies capable of inhibiting SARS-CoV-2 infectivity in vitro. 44 linear S-derived peptides were genetically fused with the RAD scaffold (RAD-SCoV-epitopes) and screened for antigenicity with sera collected from COVID-19-infected patients. In a second step, selected RAD-SCoV-epitopes were used to immunize mice and generate antibodies. Phenotypic screening showed that some of these antibodies were able to recognize replicating viral particles in VERO CCL-81 and most notably seven of the RAD-SCoV-epitopes were able to induce antibodies that inhibited viral infection. Our findings highlight the RAD display system as an useful platform for the immunological characterization of peptides and a potentially valuable strategy for the design of antigens for peptide-based vaccines, for epitope-specific antibody mapping, and for the development of antibodies for diagnostic and therapeutic purposes.

## Introduction

The emergence of new viruses with epidemic potential represents a serious threat for the society, both for human health and for economic growth. As recently observed during the COVID-19 pandemic, rapid research into host–pathogen interactions was crucial for the fast development of diagnostic methods, therapies and vaccines. Indeed, knowledge about structural and immunological features of virus proteins led very rapidly to the development of safe and effective vaccines^1^. The SARS-CoV-2 genome encodes for four structural proteins: the nucleocapsid (N) protein and the small envelope (E), membrane (M) and spike (S)^2^. The S glycoprotein is composed of two subunits: S1 with the receptor-binding domain (RBD), responsible for the recognition and binding to the target cells, and S2 that mediates the fusion of the viral membrane with the host cell^3^. Due to the importance of the S protein for the viral infection, it is the main target for vaccines, including those based on messenger RNA, adenovirus vectors, and purified proteins^4^.

Detailed understanding of the viral epitopes that are directly involved with host cells will facilitate the development of both effective vaccines and specific diagnostic methods. Peptide display systems can help to probe protein–protein interactions and represent useful technological platforms for the identification of peptide epitopes capable of inducing protective immune responses in vaccinated individuals^5^. Peptide display systems are based on the fusion of target sequences into selected protein scaffolds, derived from different microorganisms, such as phage or bacteria, which confers flexibility and stability to peptide sequences allowing evaluation interaction with receptors, small molecules, or antibodies^5,6^. Such recombinant chimeric peptides may show superior properties regarding comparison to purified synthetic peptides, complexed or not with polymers or nanoparticles, regarding immunological features of the target peptide sequence, such as antigenicity (ability to be react with antibodies raised in infected persons) and immunogenicity (ability to generate antibodies that can bind to the protein expressed on virus particles).

The archaeal peptide display system (RAD display) was developed by Rossmann and collaborators^7^ as a multipurpose scaffold system for studies of protein–protein interactions and protein function. Based on the monomeric ATPase domain of the *Pyrococcus furiosus* RadA protein, the system allows the presentation of target peptides, protein domains or even full-length proteins on a thermostable scaffold protein that is compatible with functional assays. In addition, these proteins can be rapidly expressed in *Escherichia coli* and purified easily at low costs, and with limited specialized equipment compared to other strategies such as solid-phase peptide synthesis.

In the present study, we evaluated the use of the RAD display system for probing immunological features of peptides derived from the SARS-CoV-2 Spike (S) protein. S-derived peptides were expressed in the RAD display system and probed in ELISA assays with sera collected from SARS-CoV-2 infected patients. Subsequently, the chimeric proteins were used to generate peptide-specific antibodies following immunization of mice, and, finally, the virus-neutralization activity of these mouse-derived antibodies was evaluated using live SARS-CoV-2 in cell culture. This present study demonstrates that the RAD display system represents a simple and accessible platform to assess immunological features of peptides derived from the SARS-CoV-2 virus and may be a valuable strategy for the development of serological tests, epitope-specific antibody mapping, and peptide-based vaccines in a scalable way.

## Results

### Selection and expression of SARS-CoV-2 S-derived peptides using the RAD display platform

**Figure 1:**
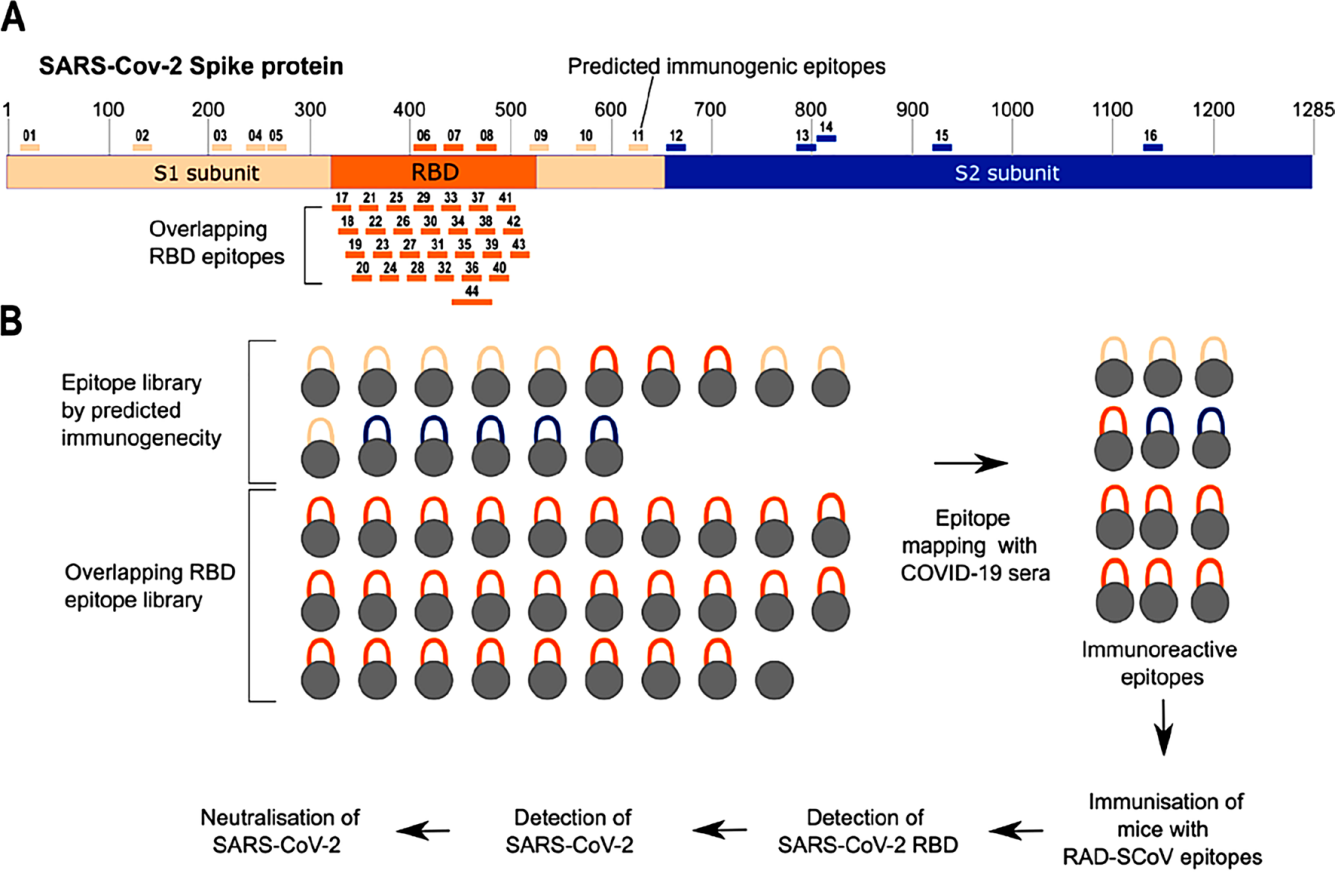
Schematic representation of the selected epitopes and workflow used for evaluation of RAD-SCoV epitopes. (**A**) The SARS-CoV-2 S protein is represented by the S1 and S2 subunits in orange and blue, respectively. The receptor-binding domain (RBD) in S1 is highlighted. Positions of expressed epitopes are indicated with small bars. (**B**) Schematic workflow of production of RAD-SCoV-epitopes and their analysis by ELISA, fluorescence microscopy and virus neutralization assays.

In silico analyses of the S protein structure and data retrieved from the Epitope Database Analysis Resource (IEDB) were used to predict immunological and structural features of peptides. Based on this analysis, we selected 15 peptides (ten of which were from S1 subunit and five from S2 subunit) to be displayed on the RAD system (Fig. 1A, RAD-SCoV-01 to -15). In addition, we designed a panel of 27 overlapping peptides with 21 amino acid residues that were separated by seven residues from the previous one (RAD-SCoV-16 to -43), covering the complete receptor binding domain (RBD) plus an additional longer epitope covering most of the ACE2 binding site (RAD-SCoV-44). The composition of our panel of RAD-SCoV-epitopes and the workflow we used to determine the antigenicity of the epitopes, to evaluate their immunogenicity and to perform the functional analyses of the RAD-SCoV-epitope-induce antibodies elicited in mice is schematically represented in Fig. 1. Sequences of all the selected 44 epitopes are listed in Supplementary Table S1.

Nucleotide sequences corresponding to the selected peptide epitopes were cloned into the phcRAD1 vector using ligation independent cloning with overlapping oligonucleotides^7^. These constructs were transformed to *E. coli* BL21(DE3) cells for expression. All RAD constructs, containing a N-terminal His-tag, were expressed in soluble form and purified to homogeneity with a combination of immobilized metal affinity chromatography (IMAC) and size-exclusion chromatography (Supplementary Figure S1).

### The RAD-SCoV-epitopes were recognized by antibodies from COVID-19-infected individuals

**Figure 2:**
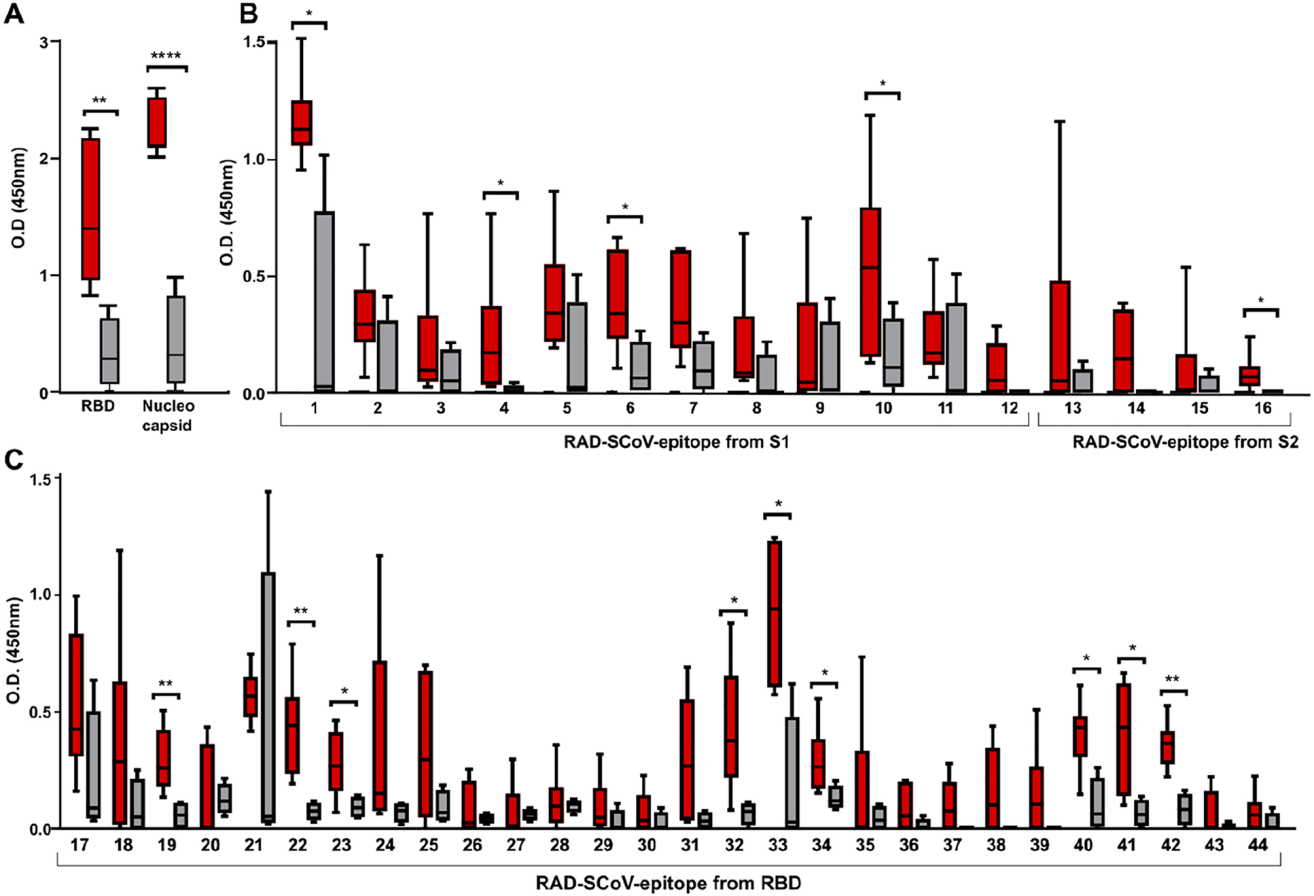
Recognition of the RAD-SCoV-epitopes by antibodies from convalescent individuals with COVID-19. ELISA plate wells were coated with 800 ng of each purified RAD-SCoV-epitope followed by incubation with 1:100 diluted human serum samples. Six sera from individuals positive for COVID-19 (red bars) and three negative samples (grey bars) were used in the assays. (**A**) Confirmation of COVID-19 status of the sera using recombinant SARS-CoV-2 nucleocapsid and RBD proteins. (**B**) Analysis of 16 epitopes selected through informatic analyses. (**C**) Analysis of the epitopes from the RBG array. All values represent the average of three tests with the results for empty RAD scaffold deducted. Statistical significance was evaluated for each RAD-SCoV-epitope against SARS-CoV-2 positive and negative sera, using Mann Whitney test. *P value: < 0.05.

RAD-SCoV-epitopes were probed as immobilised solid phase bound antigens in ELISA using serum samples from individuals exposed to the SARS-CoV-2 (n = 6), and individuals not exposed to the virus (n = 3). Recombinant RBD and the nucleocapsid (N) proteins were used to confirm COVID-status of the tested serum samples (Fig. 2). The results showed that the antigenicity of the epitopes cloned in the RAD scaffold are preserved, especially when considering that epitopes correspond to ca. 10% of the chimeric proteins and only a small fraction of the full S protein. When compared with the results observed for soluble RBD, which showed absorbance at 450 nm between 1 and 2 AU_450_, the best epitopes exposed on RAD system resulted in absorbances between 0.5 and 1 AU. Most of the recombinant RAD-SCoV-epitopes were recognized by at least one serum sample from SARS-CoV-2 infected patient, while sera collected from non-infected individuals showed low reactivity both with the empty RAD scaffold and with the chimeric RAD-SCoV-epitopes. In general, epitopes covering S-RBD were recognized by all SARS-CoV-2-positive sera. These experiments demonstrated that the S-derived epitopes expressed on RAD scaffold preserve, at least partially, the antigenicity of the epitopes present in the native virus protein.

### Induction of anti-SARS-CoV-2 antibodies in mice immunized with RAD-SCoV-epitopes

The immunogenicity of 19 selected recombinant RAD-SCoV-epitopes was evaluated in C57BL/6 mice by combination with a non-toxic derivative of the heat labile toxin as adjuvant. Mice were inoculated subcutaneously with three doses of the antigens with an interval of 14 days. Serum samples were collected 14 days after the last immunization and analysed by ELISA using the corresponding chimeric antigen as the solid phase. All sera from RAD-SCoV-epitope-immunized mice showed high antibody titres against the immunogens, including the samples from mice immunised with RAD scaffold (Supplementary Figure S2).

**Figure 3:**
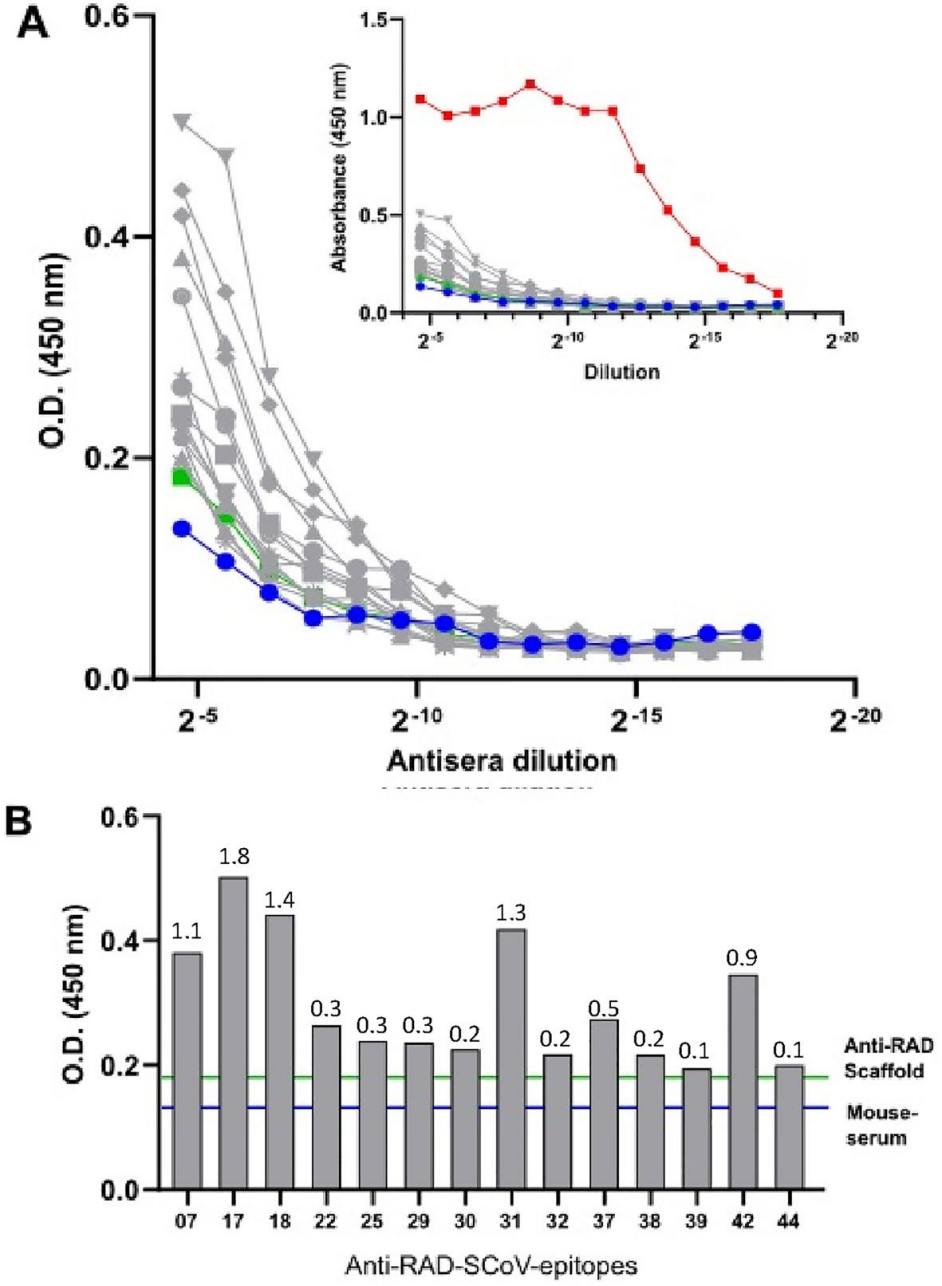
Anti-RAD-SCoV-epitope antibodies recognise purified recombinant RBD. (**A**) The end-point titration ELISA of the anti-RAD-SCoV-epitopes antibodies against the purified S protein RBD. Blue points correspond to titration with unimmunized mouse serum and the green points are data for RAD scaffold without an epitope. Insert shows the same data with additional curve for end-point titration using serum for mouse immunized with full-length RBD for comparison (**B**) Absorbance values from 1/25 dilution from the end point ELISAs performed with the anti-RAD-SCoV-epitopes produced in mouse C57BL/6 against RBD. The green line indicated the reading for RAD scaffold-immunized mouse and blue line the non-immunized mouse serum. Fold change values for each epitope compared to the anti-RAD-scaffold is shown above the columns. Statistical significance also was evaluated with the five lower serum dilutions (1:25 to 1:400) using two-way ANOVA followed by Dunnett correction (compared to the Anti-RAD-Scaffold).

We then evaluated the ability of these antisera to recognise their respective epitopes in the context of the viral proteins. To do this, we repeated the ELISA assays using recombinant RBD as the solid phase capture agent. Antibodies from all the RAD-SCoV-epitope-immunised mice reacted with the recombinant RBD and showed enhanced reactivity against the purified protein, in comparison to serum from a mouse immunised with empty scaffold or to a pre-immune serum (Fig. 3A and Supplementary Table S2). Notably, anti-RAD-SCoV-07, anti-RAD-SCoV-17, anti-RAD-SCoV-18, anti-RAD-SCoV-31 and RAD-SCoV-42 showed the highest anti-RBD titres (Fig. 3B). Taken together, the results demonstrated that all tested peptides were immunogenic in mice and stimulated production antibodies capable of recognizing SARS-CoV-2 S protein. Statistical significance evaluated for serum dilutions 1:25 to 1:400 using two-way ANOVA followed by Dunnett correction (compared to the Anti-RAD-Scaffold) showed that none were significantly different to each other.

### Anti-RAD-SCoV-epitope antibodies recognize SARS-CoV-2 virus particles

**Figure 4:**
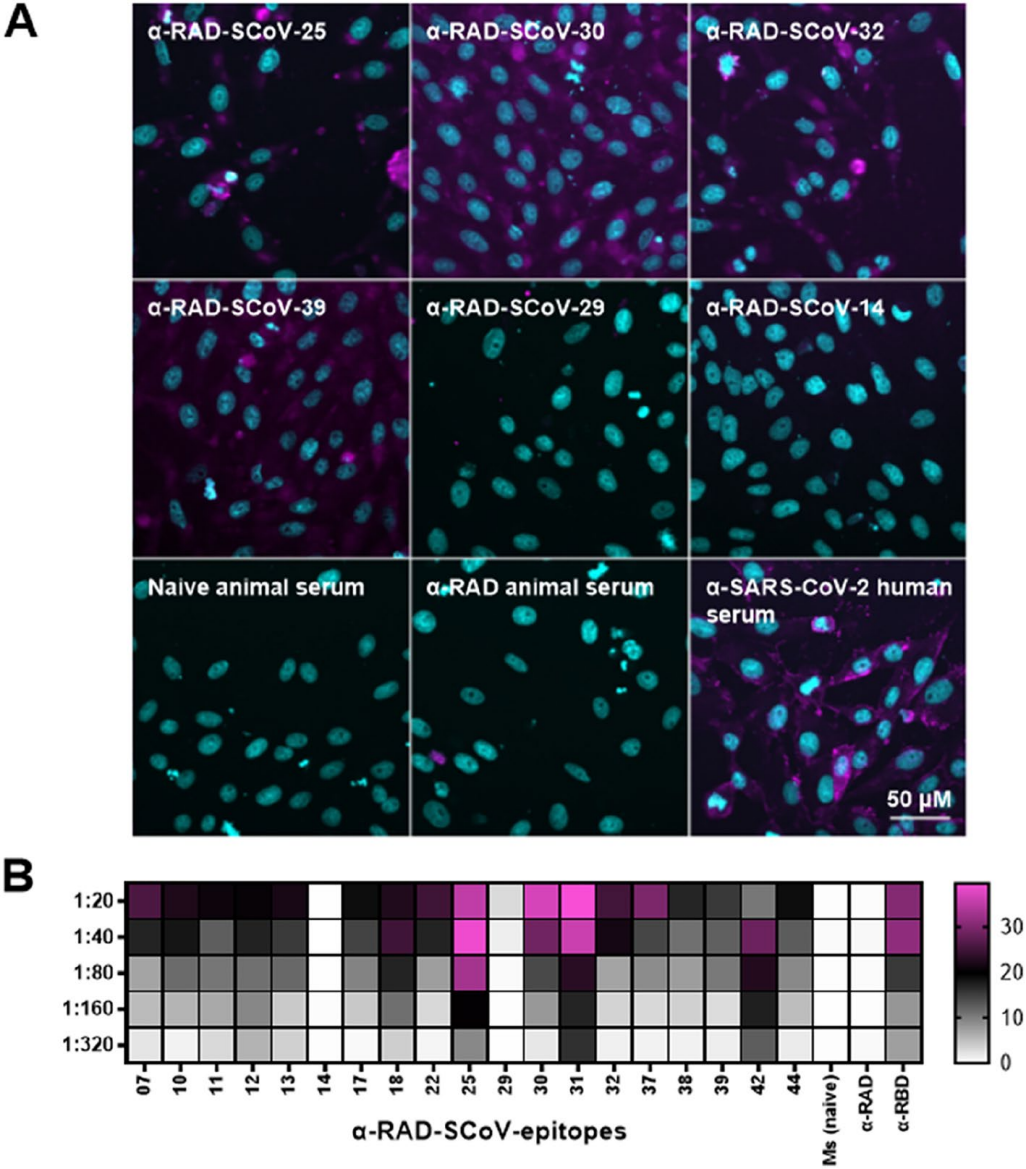
Immunofluorescence of VERO CCL-81 cells infected with SARS-CoV-2 using sera from mice immunized with the RAD-SCoV-epitopes. 1 × 10^4^ VERO cells were infected with SARS-CoV-2 at MOI of 0.02 and grown for 72 h, after which the cells were fixed and stained with indicated antisera and Alexa Fluor 488-labelled secondary antibody as well as with DAPI. Images were captured on InCell Analyser 2200 systems and images were analyzed on InCarta software. (**A**) Example images of positive and negative staining, as indicated in each panel. (**B**) Heat map of the quantified fluorescence for each antibody tested in the different dilutions showed as percentage of non-infected cells.

To evaluate whether the antibodies present in sera of the mice immunized with RAD-SCoV-epitopes were capable of recognizing SARS-CoV-2 particles in vitro, we performed immunofluorescence assays with infected VERO CCL-81 cells. From the 19 anti-RAD-SCoV-epitopes tested, anti-RAD-SCoV-07, 22, 25, -30, -31, -32, -37, and -42 were most effective in staining the viral S-protein in this assay. Anti-RAD-SCoV-10, -11, -12 and -17 presented weak immunofluorescent staining at 1:20 dilution while the remaining anti-RAD-SCoV-15, -29, -38 and -39 sera were not able to recognize the viral proteins (Fig. 4A and Supplementary Figure S3). Antibodies against RAD itself did not bind to virus-infected cells. The best results were obtained with antibodies raised against epitopes from RBD part of S protein. Quantification of the immunofluorescence for each antibody is shown as a heatmap in Fig. 4B. These data demonstrated that SARS-CoV-2 epitopes displayed in the RAD scaffold can induce specific humoral immune responses with antibodies capable of recognizing the viral S protein.

**Figure 5:**
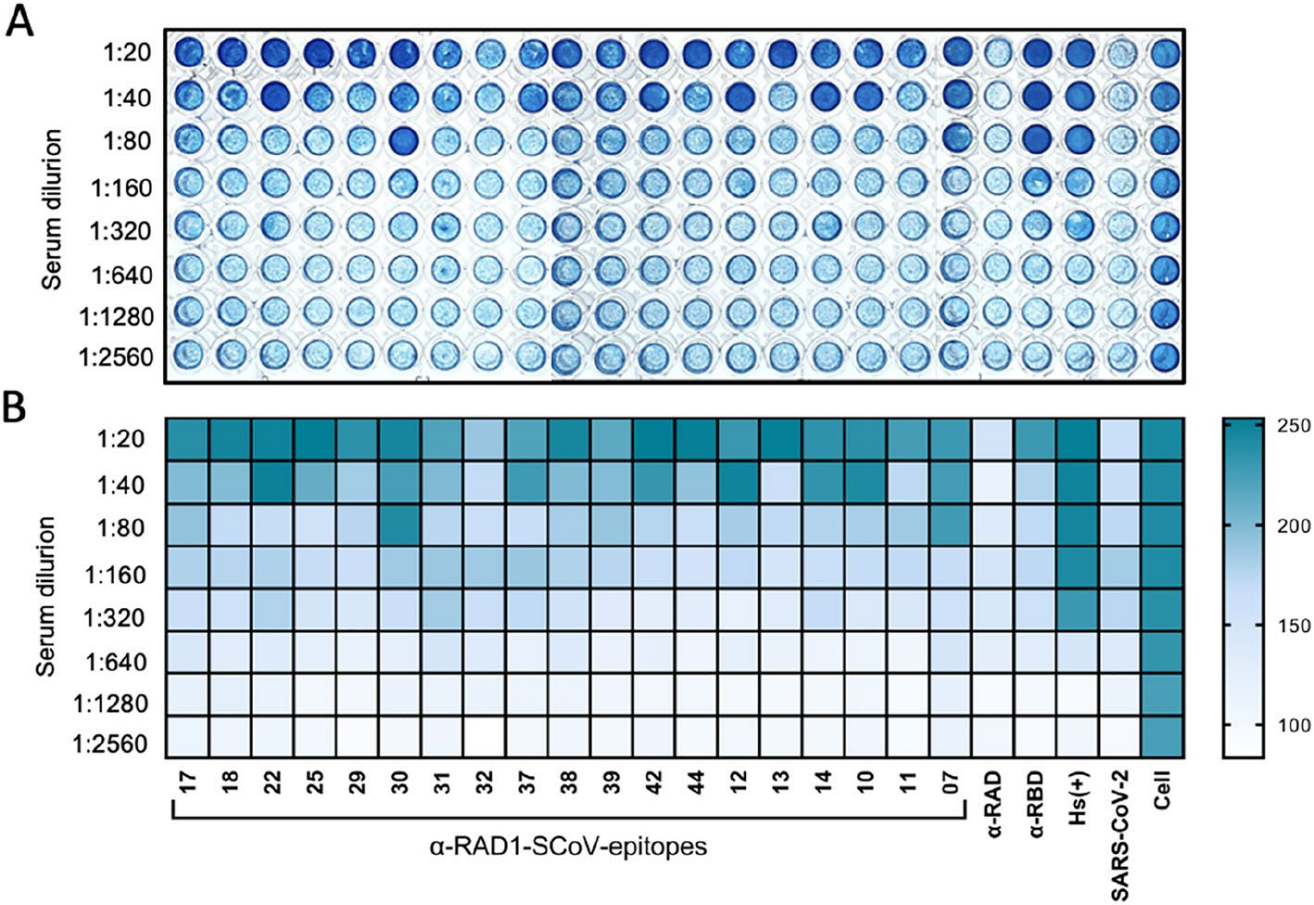
Analysis of SAR-CoV-2 neutralising activity of anti-RAD-SCoV-epitope mouse sera. (**A**) Virus neutralization test (VNT) with the anti-RAD-SCoV-epitope sera. Samples were tested for neutralising antibodies using the cytopathic effect‐based virus neutralisation test (CPE‐VNT) using a SARS-CoV-2 (original Wuhan isolate). VERO CCL-81 cells with strong blue staining indicates uninfected cells. Panel (A) is a composite image from the original data, with samples ordered numerically. Original images are shown in Supplementary Figure S4. (**B**) Heatmap representation of the results for each antibody according to neutralization at indicated dilutions.

Antibodies against RAD-SCoV-epitopes can neutralize SARS-CoV-2. The sera obtained from the immunized mice were also tested for the presence of neutralizing antibodies using the cytopathic effect‐based virus neutralization assay (CPE‐VNT) described in Wendel and collaborators^8^ using a Wuhan variant of SARS-CoV-2. Virus neutralization titres, referred to as VNT100, are described as the highest dilution of serum that neutralized virus growth and which was evident by the absence of cytopathic effect (dark blue; Fig. 5). The results of this assay showed that at the 1:20 dilution all the sera, except for anti-RAD-SCoV-32, could neutralize the virus. Anti-RAD-SCoV-07 and anti-RAD-SCoV-30 sera showed the best results, with inhibition of cytopathic effects at 1:80 dilution (Fig. 5 and Supplementary Figure S4). These results are in agreement with the immunofluorescence assays which demonstrated the ability of these antibodies to recognise epitopes exposed on virus particles.

## Discussion

**Figure 6:**
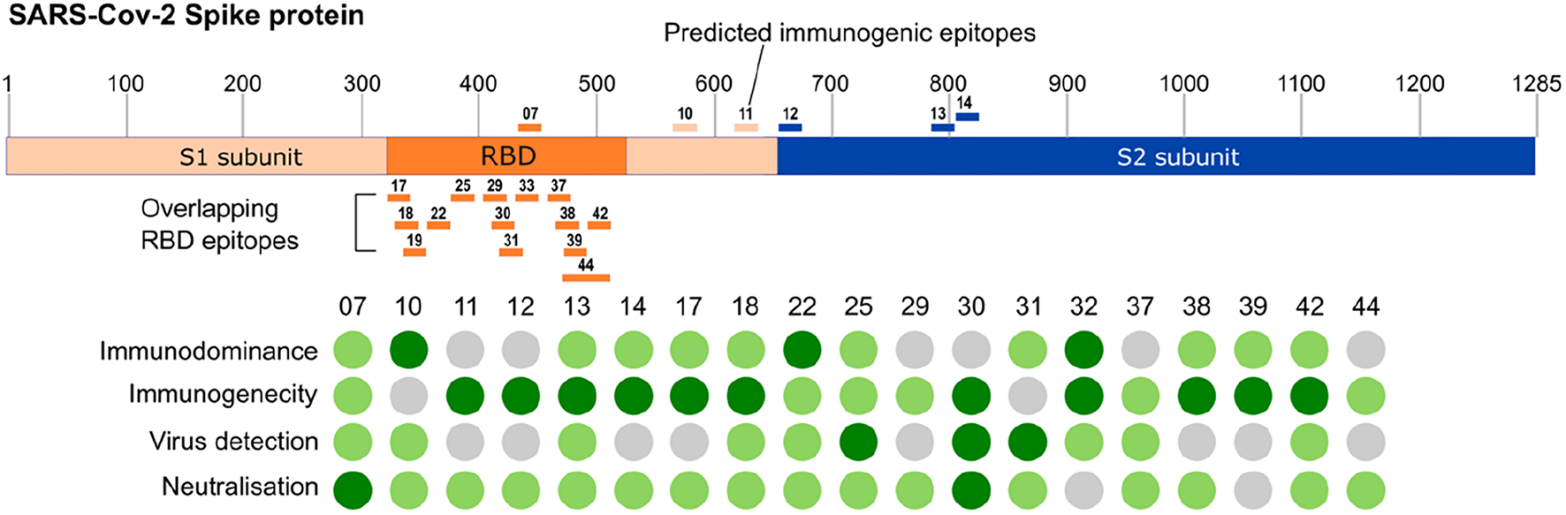
Summary of immunological features associated with RAD-SCoV-epitopes analysed in the present study. Initially, 44 RAD-SCoV-pep chimeric proteins were generated after insertion of SARS-CoV-2 derived peptides on the RAD-Scaffold and tested with sera of COVID-19 infected individuals (immunodominance). From the constructs with preserved antigenicity, 19 were inoculated in C57BL/6 black mice to evaluate the capacity to induce specific antibodies (immunogenicity). Antibodies raised against the peptide epitopes were screened for the ability to bind the S protein expressed on cells infected with SARS-CoV-2 (virus detection) and, subsequently, for the capacity to neutralize infection of the virus using VERO CCL-81 cells (neutralization). The performance of each peptide/antibody is summarized with the green dots with the darker color indicating stronger response in the respective assay.

We have demonstrated in this study that the RAD display system is an effective tool for determining the antigenicity and immunogenicity of microorganism-derived peptides. We used this approach to screen specific SARS-CoV-2 S protein peptides and observed that several peptides displayed in the RAD system were recognized by human sera and elicited neutralizing antibodies in mice after immunization. Among the four structural proteins of SARS-CoV-2, the Spike (S) protein plays a central role in virus entry and replication. The immunogenicity, immunodominance and neutralisation capacity of several epitopes of S protein have been extensively reported^9,10^. There has been a significant effort to map the immunodominance landscape of the S protein epitopes, including the effect of emerging mutations in SARS-CoV-2 that might alter the antigenicity of the virus and affect human immune responses. Most of the published studies have been using synthetic peptides^11^, exhibition on HBc-Spy-Catcher (HBc-S), VLPs^12^ and phage display approaches^13^. Production of synthetic peptides requires either expensive specialist hardware or is costly when done externally. It is hard to predict the solubility and stability of isolated peptides and their use can be significantly affected by their properties, such as hydrophobicity and charge. Alternatively, in this work, we evaluated the use of the RAD display system based on the *P. furiosus* RadA protein as a scaffold for the exhibition of peptides tested for recognition by antibodies present in sera from COVID-19 positive individuals and the generation of neutralizing antibodies. The use of RAD display is particularly suitable once the protein was produced in large amounts, soluble, and in a highly stable form. Presenting the peptides in a partially constrained for in the background of otherwise soluble, larger proteins should also partly protect them from possible proteolysis as well as “buffering” the differences between the physico-chemical properties of the epitopes in subsequent assays. Forty-four epitopes from SARS-CoV-2 S protein were expressed as fusion chimeric proteins in the RAD display system and evaluated for immunological features. They were, first, evaluated for antigenicity and immunodominance, with sera collected from SARS-CoV-2 infected patients, and 19 were, subsequently, used for immunogenicity analyses and virus neutralization activity of sera collected from inoculated mice. At least 19 peptides exposed on the RAD display generated antibodies capable to interfere with the infection of VERO CCL-81 cells by SARS-CoV-2 at in vitro conditions. From these, 14 were from RBD region, two from S1 (not RBD) and three from S2 region (Fig. 6). These results were in accordance with previous studies, based on different approaches to identify SARS-CoV-2 epitopes capable to generate antibodies with virus-neutralizing activity^9,13^. Thus, RAD scaffold represents a promising alternative for the expression of epitopes with preserved immunological features both for interaction with antibodies raised during natural infections and to generate specific antibodies able to recognize the corresponding sequences on the virus protein.

Peptides exposed in RAD display were mostly recognized by antibodies present in sera of, at least, three PCR-confirmed SARS-CoV-2 infected convalescent patients (total of six). Considering that the peptides correspond to ca. 10% of the chimeric RAD protein, the results, plotted for the peptides in comparison with the control samples, purified RBD, and the Nucleocapsid protein, were significant. The best results were obtained for peptides from RBD (even if not necessarily within the ACE2 interface) and S2, which were recognized by more than three sera with absorbance values of over 1.0 AU in the ELISA. There was, however, no clear consensus for the recognition of the peptides by the antibodies present in the different sera. In fact, the immune responses to SARS-CoV-2 infection in convalescent individuals are characterized by the production of a diverse range of antibodies targeting different regions of the virus. This diversity is important, as it allows the immune system to recognize and neutralize a variety of viral strains and variants. In the case of SARS-CoV-2, the RBD has been described as the most relevant antigen target for antibodies and represents a critical region of the virus responsible for binding to the ACE2 receptor on human cells and mediating viral entry into host cells.

Antigenicity and immunodominance are not necessarily linked to immunogenicity. Immunogenicity refers to the ability of an antigen, included or not into a vaccine formulation, to induce specific immune responses. As shown by our data, most of the S protein-derived peptides were clearly immunogenic. All the RAD-SCoV-epitopes used for the immunization of mice were able to stimulate immune response and to induce production of antibodies against the epitopes (Supplementary Figure S2). Although the induced antibody responses were partially directed against the RAD protein, there were also antibodies capable to recognize recombinant RBD protein under in vitro conditions (Fig. 3).

The antibodies anti-RAD-SCoV-epitopes also were able to recognize the virus particles, such as demonstrated in fluorescence microscopy experiments, and some were capable to interfere with the ability of virus particles to infect susceptible host cells kept under in vitro cultivation. The highest binding levels were obtained with antibodies raised in mice immunized with RAD-SCov-07 and RAD-SCov-30, localized in S1 subunit, particularly in the RBD, but antibodies raised in mice inoculated with other RAD-SCoV-epitopes containing peptides from the RBD region also showed promising results. These results demonstrated that different epitopes derived from the S protein are targets for antibodies capable of blocking the infection and confirm, mainly based on the generation of monoclonal antibodies, that antibodies targeting RBD, S1 and S2-derived peptides are commonly found in convalescent individuals and effectively neutralize the virus^11^. While the RBD is the most relevant antigen target, antibodies binding to S2 subunit epitopes, as well as other regions of the virus S protein also play a relevant role in providing protection against the virus^10^. In line with this, antibodies raised against epitopes in S1 domain but outside RBD (RAD-SCoV-10, -12 and -15) interfered with the infection capacity of SARS-CoV-2.

Most of the SARS-CoV-2 S-derived epitopes used in our work have been reported previously as being immunogenic either in vitro, in vivo or in silico^11,13,14^. In silico analyses have revealed that the amino acid sequences contained in RAD-SCoV-22 (^368^LYNSASFSTFKCYGVSPTKLN^389^) and RAD-SCoV-29 (^417^KIADYNYKLPDD FTGCVIAWN^438^) as potential candidate for vaccinal epitopes thanks to their location in the interface of RBD and ACE2^14,15^. These regions contain a readily mutated residue (G382), detected among different variants of concern, which is recognized by HLA molecules^16^. Moreover, the sequence covering residues 368 to 438 overlaps with CR3022’s epitope. CR3022 was previously isolated from a convalescent SARS patient, and it is a neutralizing antibody that targets the receptor binding domain (RBD) of SARS-CoV^16^. It targets a highly conserved epitope, distal from the receptor binding site, that enables cross-reactive binding between SARS-CoV-2^17^. A recent study has shown that CR3022 can also bind to the RBD of SARS-CoV-2^16^. This epitope stimulates robust secretion of IFN-γ from splenocytes and is detected by more than 40% of the sera from SARS-CoV-2 infected patients^18^. Also, epitopes present in this region were shown to be recognized by CD8^+^ T cells of patients with COVID-19^19^ (Table 1). Peptides that cover most part of RAD-SCoV-18 (sequence ^340^LYNSASFSTFKCYGVSPTKLN^361^) have been described to induce CD8^+^ T-cells in previous studies^18,20,21^. Sequences that cover RAD-SCoV-30 and RAD-SCoV-31 (^424^KLPDDFTGCVIAWNSNNLDSK^445^ and ^431^GCVIAWNSNNLDSKVGGNYNY^452^, respectively) were previously demonstrated to be recognized by antibodies (hNAbs)^22^ and to induce neutralizing antibodies^18,21^. RAD-SCoV-42 (^508^YRVVVLSFELLHAPATVCGPK^529^) is recognized by the antigen presenting cells MHC-I molecules^18,23^ and the peptide covering the sequence of the RAD-SCoV-07 (^442^DDSKVGGNYNYLY^452^), and part of RAD-SCoV-30 and RAD-SCoV-31 (^424^KLPDDFTGCVIAWNSNNLDSK^445^ and ^431^GCVIAWNSNNLDSKVGGNYNY^452^, respectively) can induce antibodies capable of neutralizing the virus^18,21,22^ (Table 1). RAD-SCoV-13 and RAD-SCoV-15 contain immunodominant epitopes that induced antibodies that are capable of inhibiting SARS-CoV-2 pseudoviruses^24,25^. and sequences within RAD-SCoV-07, RAD-SCoV-10 and RAD-SCoV-13 hold SARS-CoV-2 neutralising epitopes, as demonstrated by immunoinformatic mapping tools^22^. Additional references for previous characterisation of our epitopes are listed in Table 1.

**Table 1:** Validation of the results obtained with RAD display.

RAD-SCoV-epitope	Amino acids	Epitope sequence	References
RAD-SCoV-07	442–452	DSKVGGNYNYLY	^22^
RAD-SCoV-10	568–587	FGRDIADTTDAVRDPQTLEI FGRDIADTTDAVRDPQTLEI FGRDIADTTDAVRDPQTLEI FGRDIADTTDAVRDPQTLEI	^16,26–28^
RAD-SCoV-11	628–647	QLTPTWRVYSTGSNVFQTRA	^16^
RAD-SCoV-12	668–687	PIGAGICASYQTQTNSPSGAGSV	^24^
RAD-SCoV-13	788–807	VKQIYKTPPIKDFGGFNFSQ VKQIYKTPPIKDFGGFNFSQ VKQIYKTPPIKDFGGFNFSQ VKQIYKTPPIKDFGGFNFSQ VKQIYKTPPIKDFGGFNFSQ	^12,24,25,27,29^
RAD-SCoV-15	1137–1148	VYDPLQPELDSF	^22^
RAD-SCoV-17	333–354	TNLCPFGEVFNATRFASVYAW TNLCPFGEVFNATRFASVYAW	^22,23^
RAD-SCoV-18	340–361	EVFNATRFASVYAWNRKRISN EVFNATRFASVYAWNRKRISN	^22,23^
RAD-SCoV-22	368–389	LYNSASFSTFKCYGVSPTKLN LYNSASFSTFKCYGVSPTKLN LYNSASFSTFKCYGVSPTKLN	^16,20,21^
RAD-SCoV-25	389–410	DLCFTNVYADSFVIRGDEVRQ DLCFTNVYADSFVIRGDEVRQ	^22,23^
RAD-SCoV-29	417–438	KIADYNYKLPDDFTGCVIAWN KIADYNYKLPDDFTGCVIAWN	^18,23^
RAD-SCoV-30	424–445	KLPDDFTGCVIAWNSNNLDSK KLPDDFTGCVIAWNSNNLDSK KLPDDFTGCVIAWNSNNLDSK	^18,21,22^
RAD-SCoV-31	431–452	GCVIAWNSNNLDSKVGGNYNY GCVIAWNSNNLDSKVGGNYNY GCVIAWNSNNLDSKVGGNYNY	^18,21,22^
RAD-SCoV-33	445–466	VGGNYNYLYRLFRKSNLKPFE VGGNYNYLYRLFRKSNLKPFE VGGNYNYLYRLFRKSNLKPFE VGGNYNYLYRLFRKSNLKPFE	^22,28,30,31^
RAD-SCoV-37	473–494	YQAGSTPCNGVEGFNCYFPLQ YQAGSTPCNGVEGFNCYFPLQ YQAGSTPCNGVEGFNCYFPLQ YQAGSTPCNGVEGFNCYFPLQ	^21,22,28,32^
RAD-SCoV-38	480–501	CNGVEGFNCYFPLQSYGFQPT CNGVEGFNCYFPLQSYGFQPT CNGVEGFNCYFPLQSYGFQPT	^22,32,33^
RAD-SCoV-39	487–508	NCYFPLQSYGFQPTNGVGYQP NCYFPLQSYGFQPTNGVGYQP NCYFPLQSYGFQPTNGVGYQP	^22,23,34^
RAD-SCoV-42	508–529	YRVVVLSFELLHAPATVCGPK YRVVVLSFELLHAPATVCGPK	^18,23^
RAD-SCoV-44	452–494	LYRLFRKSNLKPFERDISTEIYQAGSTPCNGVEGFNCYFPLQ LYRLFRKSNLKPFERDISTEIYQAGSTPCNGVEGFNCYFPLQ LYRLFRKSNLKPFERDISTEIYQAGSTPCNGVEGFNCYFPLQ LYRLFRKSNLKPFERDISTEIYQAGSTPCNGVEGFNCYFPLQ LYRLFRKSNLKPFERDISTEIYQAGSTPCNGVEGFNCYFPLQ	^21,22,25,26,30^

Collectively, the present results validated the use of RAD display system as an approach for expression of peptides for immunological studies, from serological diagnostic methods to the generation of monospecific polyclonal antibodies capable to interfere with the infectivity of viruses. Most of the tested peptide sequences, expressed as chimeric proteins genetically fused with the RAD scaffold, displayed some immunological features, ranging from recognition by antibodies induced after SARS-CoV-2 infection to the generation of antibodies capable to bind and neutralize the infectivity of virus particles. Thus, the peptide display strategy represents a useful technological platform with the potential to contribute to the understanding of immunological features of antigens derived from different microorganisms and contribute for the development of both diagnostic methods and prophylactic/therapeutic strategies for different pathogens. It is worth to address that while the RAD scaffold is a powerful tool for expression of peptides in *E. coli*, the host lacks post translational modifications (PTM) found in eukaryotes. For the epitopes that require PTMs, such as glycosylation, to stimulate antibodies that recognize the native target protein, this system is unlikely to work as expected.

## Material and methods

All methods were performed in accordance with the relevant guidelines and regulations.

### Animal statements

All reported experiments and protocols on live vertebrates were approved by the Research and Ethics Committee of the University of São Paulo, Institute of Biomedical Sciences (CEUA, https://ww3.icb.usp.br/ceua/) according to protocol number: 6600060820. All experiments were performed in accordance with the Brazilian Federal laws 11.794 that establishes procedures for the scientific use of animals and human studies and in State Law nº 11.977 that establishes the Code of Protection to Animals of the State of São Paulo. All methods were reported in accordance with ARRIVE guidelines (https://arriveguidelines.org). Female wide-type (WT) C57BL/6 mice of 6 weeks old were obtained from Central Animal House of the Faculty of Medicine of the University of São Paulo and maintained in pathogen-free conditions according to the guidelines for animal care (https://biot.fm.usp.br/index.php?mpg=02.01.00).

### Human sera

Informed consent forms for use of samples were obtained from each patient. The protocols and consents were included in the previously approved by the Research and Ethics Committee of the University of São Paulo (CEUA), Institute of Biomedical Sciences under protocol number 6600060820 and Platform Brasil (https://conselho.saude.gov.br/plataforma-brasil-conep?view=default) protocol number 45834621.2.0000.5467, prior to study implementation. Serum samples were obtained from patients not requiring hospitalization with active or previous SARS-CoV-2 infection confirmed by RT-PCR.

### Choice of epitopes, bioinformatics and cloning into RAD display

The SARS-CoV-2 S protein amino acids sequence was obtained from the National Centre for Biotechnology Information (NCBI) and submitted to the Immune Epitope Database and Analysis Resource (IEDB) (https://www.iedb.org/) for prediction and analysis of the sites of B cells recognition and immunogenic sequences. In total, 44 peptide sequences covering the full S protein were defined and mapped in its structural model (PDB: 5I08). The corresponding nucleotide sequences of each peptide were used to design the oligonucleotides for cloning into the phRAD vector according to Rossmann and collaborators^7^. The list of oligonucleotides is presented in the Supplementary Table S3.

### Expression and purification

The plasmids containing the inserts were transformed into *E. coli* BL21(DE3) cells containing the pUBS520 plasmid which carries the tRNA for a rare arginine codons AGA/AGG. The cells were transformed using 50 ng of DNA by heat shock and grown on LB-agar plates containing ampicillin (100 μg/ml) and kanamycin (25 μg/ml) for 12 to 15 h at 37 °C. A pre-inoculum was performed with the colonies and subsequently added to 2xYT medium at 37 °C with shaking at 200 rpm until it reached an optical density at 600 nm (OD) of 0.6, after which 0.4 mM isopropyl β-D-1-thiogalactopyranoside (IPTG, Sigma-Aldrich) was added. The culture was then incubated at 18 °C with 200 rpm shaking for 8 h. The cells were centrifuged at 4032 g for 10 min and transferred to a buffer containing 50 mM Tris–HCl, 150 mM NaCl, SigmaFast Protease Inhibitor (Sigma-Aldrich), 1 μg lysozyme (Sigma-Aldrich), 10 μg/ml DNaseI (Thermo Scientific) and 10 mM magnesium chloride. Cell lysis was carried out by sonication for a total of 12 min, with 24 cycles of 30 s sonication and 30 s pause. The cell lysate was centrifuged at 25200 g for 60 min. The soluble fraction was filtered through a 0.22 μm filter and subjected to nickel affinity chromatography on a 300 μl Ni Sepharose 6 Fast Flow resin (Cytiva Life Sciences). The resin was pre-equilibrated with 50 mM Tris–HCl, 150 mM NaCl, pH 8 buffer in Poly-Prep chromatography columns (BioRad). After equilibration, the soluble extract was added. Two washes were performed, the first containing the initial buffer supplemented with 10 mM Imidazole and the second containing the initial buffer supplemented with 20 mM Imidazole. Recombinant proteins were eluted using 300 mM Imidazole. The eluted samples were concentrated in an Amicon concentrator tube, with an exclusion cutoff of 10,000 MW (Amicon, Millipore), desalted on Sephadex G-25 columns (Cytiva Life Sciences), and subjected to size exclusion chromatography on HiLoad 16/600 Superdex 75 pg column (Cytiva Life Sciences).

### Evaluation of recognition of peptides by antibodies present in human sera from patients infected with SARS-CoV-2

ELISA plates (Corning High Binding 9018) were coated using 800 ng each purified RAD-SCoV-pep in carbonate buffer pH 9.6 and incubated per 1 h at 37 °C. The plates were then washed four times with PBS containing 0.05% Tween 20 (PBS-Tween) and blocked for 3 h with a buffer containing 80 mM lysine, 74 mM mannitol and 0.05% Tween 20 in PBS. The primary antibodies obtained from human sera of COVID-19 convalescent and non-infected individuals were diluted 1:100 in a 100 mM Tris base, 10 mM Casein, 0.02% Tween 20, 500 mM sodium chloride and 12 mM EDTA, and incubated at 37 °C during 1 h. After another washing for four times with PBS-Tween, the secondary antibody Anti-IgG-Human peroxidase conjugated (SIGMA) was added. The antibody was diluted 1:4,000 in a sample buffer and incubated at 37 °C during 1 h. After washing with PBS-Tween, the plates were developed using TMB single solution (Life) and the reaction stopped using 0.2 N Na_2_SO_4_.

### Immunisation of mice with the selected epitopes

C57BL/6 mice with six weeks of age were immunized with one of 19 selected RAD-SCoV-epitopes. The immunization regime consisted of three doses of 10 μg of each protein and 1 μg of the non-toxic heat-labile toxin-1 as an adjuvant, administered at 1st, 14th and 28th days^33^. 14 days after the last immunization, peripheral blood samples were collected via the submandibular plexus. Blood samples were inactivated at 56 °C for 30 min and cooled at 4 °C for 30 min. After this period, they were centrifuged at 1,008 g for 20 min and the serum was transferred to a new and sterile tube. All the sera were stored at − 20 °C.

### Western blotting

The SDS-PAGE was run using 300 μg of the S-RBD protein in a single single well and proteins transferred to a PVDF 0.22 μm membrane (Thermo Scientific). A duplicate gel was stained with Coomassie Brilliant Blue R-250. After the transfer, the membrane was blocked using TBS-BSA 5% at RT for 3 h. Mice sera were diluted 1:20 in TBS containing 5% (w/v) BSA and incubated with the membrane overnight at 4 °C. The membranes were washed 3 times with TBS, the secondary antibody anti-IgG mouse conjugated alkaline phosphatase produced in goat (Sigma-Aldrich A1418) was added (1:30,000 dilution) and incubated with the membrane during 1 h at RT. After, the membranes were washed 3 times with TBS and developed using BCIP/NBT precipitating substrate (SigmaFast, Sigma-Aldrich). Deionized water was used to wash the membrane and remove the substrate solution.

### Cell line and virus

The VERO CCL-81 cells line obtained from ATCC (https://www.atcc.org/products/ccl-81) was cultured in Dullbecco’s Modified Eagle Medium (DMEM) (Gibco, Ref: 11,965–092) supplemented with 10% heat-inactivated fetal bovine serum (FBS) (Gibco, Ref: 12,657–029). The SARS-CoV-2 (GenBank MT350282.1) were grown and titrated as previously described^35^. The experiments involving SARS-CoV-2 were performed in a biosafety level 3 laboratory (BSL-3) located at the Institute of Biomedical Sciences in the University of São Paulo.

### Immunofluorescence

VERO CCL-81 cells (1 × 10^4^) were seeded in 96-well black plates (Greiner Ref: 655,096) and 24 h later, the cells were infected with 10^2^ TCID of SARS-CoV-2 and incubated for 72 h at 37 °C/5% CO_2_. The culture medium (DMEM, 2% FBS) containing the virus was discarded and the cells were fixed with paraformaldehyde 4% (Fixative Solution—Invitrogen, Ref: FB002) for 15 min at room temperature (RT). The cells were washed with Phosphate Buffered Saline (PBS) (Gibco, Ref: 10,010–0230) and incubated with PBS, 5% Bovine Serum Albumin (BSA) for 1 h. After washing steps, two-fold serial dilutions of mice serum samples (1:20 to 1:2560) were added to the cells and the plate was incubated at 37 °C for 1 h at RT. The serum samples anti-RAD and anti-RBD were used as negative and positive controls of this assay. After washing steps, AlexaFluor 488 goat IgG anti-mouse (Invitrogen) diluted 1:1500 in PBS, 2% BSA was added into the wells for another one hour incubation. The cell nuclei were stained with NucBlue reagent (Invitrogen, Ref: R376060) according to the manufacturer's instructions. Images were acquired in the High Content Imaging InCell Analyzer 2200 (GE Healthcare, Life Sciences) from the Core Facilities to support Research (CEFAP), Institute of Biomedical Sciences, University of São Paulo, and the data was analysed using the IN Carta Image Analysis Software, v. 1.11.3667461 (Molecular Devices).

### Virus neutralization test

The cytopathic effect (CPE)-based virus neutralisation test (VNT) was performed in 96-well plates with 1 × 10^4^ VERO cells/well 24 h prior to the experiment. Previously heat-inactivated serum samples (RAD-SCoV-peps-immunised animals) were serially diluted by a factor of two (1:20 to 1:2,560). Subsequently, 10^2^ TCID of SARS‐CoV‐2 were added to the diluted serum samples and the mixture was incubated for 1 h at 37 °C/5% CO_2_. The serum and virus mixtures were transferred to the cell monolayer and incubated for 72 h at 37 °C/5% CO_2_. After this period, each well was analysed on the microscopy to evaluate the presence of cytopathic effects. Virus-containing medium was removed from the plates and the cells were fixed/stained with naphthol blue black dye (0.1% amido black solution [w/w] with 5.4% acetic acid, 0.7% sodium acetate) for 30 min at RT. The neutralizing antibody titres correspond to the highest serum dilution capable of neutralizing virus particles (absence of cytopathic effects). Uninfected and SARS-CoV-2 infected cells (no serum) were included as controls.

### Statistics

For Fig. 2, statistical significance was evaluated for each RAD-ScoV-peptide against SARS-CoV-2 positive and negative sera, using Mann Whitney test. In the Fig. 3, statistical significance was evaluated with the five lower serum dilutions (1:25 to 1:400) using two-way ANOVA followed by Dunnett correction (compared to the Anti-RAD-Scaffold) and the fold-change is shown in the graph.

### Supplementary Information

Supplementary Information.

## Data Availability

The datasets used and/or analysed during the current study are mostly presented in the Supplementary information apart the raw data from immunofluorescence assays that can be available from the corresponding author on reasonable request.
