# Supplementary material for: SARS-CoV-2 Spike protein peptides displayed in the Pyrococcus furiosus RAD system preserve epitopes antigenicity, immunogenicity, and virus-neutralizing activity of antibodies
Source: Sci Rep. 2023 Oct 5;13:16821. doi: 10.1038/s41598-023-43720-8 (PMC10556064; doi:10.1038/s41598-023-43720-8)
Supplement: Supplementary file 1 — Supplementary Information. [file 41598_2023_43720_MOESM1_ESM.pdf]

Supplementary material for

SARS-CoV-2 Spike protein peptides displayed in the *Pyrococcus furiosus* RAD system preserve epitopes antigenicity, immunogenicity, and virus-neutralizing activity of antibodies

Victor Bolsanelli Cioffi[1], Maria Fernanda de Castro-Amarante[2], Aleksei Lulla[3], Robert Andreata-Santos[2], Mario Costa Cruz[4], Ana Carolina Ramos Moreno[2,5], Mariângela de Oliveira Silva[6], Bianca de Miranda Peres[6], Lucio Holanda Gondim de Freitas Junior[6], Carolina Borsoi Moraes[6], Edison Luiz Durigon[7,8], Nicola Coker Gordon[3], Marko Hyvonen[3], Luís Carlos de Souza Ferreira[2,8], Andrea Balan[1*]

[1]Laboratory of Applied Structural Biology, Department of Microbiology, Institute of Biomedical Sciences, University of São Paulo, Av. Prof. Lineu Prestes, 1374, 05508-000, São Paulo, Brazil. [2]Laboratory of Vaccine Development, Department of Microbiology, University of São Paulo, Institute of Biomedical Sciences, Av. Prof. Lineu Prestes, 1374, 05508-000, São Paulo, Brazil. [3]Department of Biochemistry, University of Cambridge, 80 Tennis Court Road, Cambridge CB2 1GA, UK. [4]Core Facilities to support Research (CEFAP), Institute of Biomedical Sciences, University of São Paulo, Av. Prof. Lineu Prestes, 1730, 05508-000, São Paulo, Brazil. [5]Vaccine Development Laboratory, Butantan Institute, Av. Vital Brasil, 1500São

Paulo, SP, 05503-900, Brazil. [6]Phenotypic Screening Platform, Department of Microbiology, University of São Paulo, Institute of Biomedical Sciences, Av. Prof. Lineu Prestes, 1374, 05508-000, São Paulo, Brazil. [7]Laboratory of Clinical and Molecular Virology, Institute of Biomedical Sciences, University of São Paulo, Av. Prof. Lineu Prestes, 1374, 05508-000, São Paulo, Brazil. [8]Institut Pasteur de São Paulo, Av. Prof. Lucio Martins Rodrigues, 370, 05508-020, São Paulo, Brazil. *Correspondence and requests for materials should be addressed to A.B. to: (email: abalan@usp.br)

Tables

Table S1. List of Rad recombinant proteins exposing SARS-CoV-2 Spike peptides and their properties. The underlined residues were predicted as immunogenic and exposed in the three-dimensional structure of Spike, according to the Immune Epitope Database and Analysis Resource (IEDB) (https://www.iedb.org) and structural analysis.

Construction	Number amino acids	Peptide Sequences	IEDB values min-max/ average	Position
RAD-SCoV-01	13-34	TQCVNLTTRTQLPPAYTNSFTR	0.244-0.611/ 0.468	S1
RAD-SCoV-02	135-154	FCNDPFLGVYY**HKNNKS**WME	0.243-0.569/ 0.434	S1
RAD-SCoV-03	207-221	HTPINL**VRDLP**QGFS	0.208-0.553/ 0.424	S1
RAD-SCoV-04	245-267	HRSYLTPGDSSSGWTAGAAAYYV	0.228-0.641/ 0.492	S1
RAD-SCoV-05	269-285	YLQPRTFLLKY**NE**NGTI	0.234-0.507/ 0.417	S1
RAD-SCoV-06	406-432	EVRQIAPGQTGKIADYNYKLPDDFTGC	0.236-0.568/ 0.492	S1
RAD-SCoV-07	442-452	DSKV**GGNYN**YLY	0.239-0.562/ 0.426	S1
RAD-SCoV-08	465-487	ERDISTEIYQAGSTPCNGVEGFN	0.252-0.586/ 0.493	S1
RAD-SCoV-09	525-536	GPKK**STNL**VKN	0.247-0.591/ 0.437	S1
RAD-SCoV-10	568-587	FGRDIADTTDAVRDPQTLEI	0.265-0.585/ 0.490	S1
RAD-SCoV-11	628-647	QLTPTW**RVYSTGSNVF**QTRA	0/204-0.580/ 0.461	S1
RAD-SCoV-12	668-687	PIGAGIC**ASYQTQTNS**PSGAGSV	0.175-0.686/ 0.496	S1
RAD-SCoV-13	788-807	VKQIYKTPPIKDFGGFNFSQ	0.279-0.604/ 0.501	S2
RAD-SCoV-14	808-827	ILPDPSKPSKRSFIEDLLFN	0.250-0.579/ 0.472	S2
RAD-SCoV-15	936-941	DSLSST	-/-	S2
RAD-SCoV-16	1137-1148	VYDPLQPELDSF	0.241-0.533/ 0.413	S2
RAD-SCoV-17	333-354	TNLCPFGE**VFNATRFAS**VYAW	0.229-0.532/ 0.444	RBD
RAD-SCoV-18	340-361	EVFNATRFASVYAWNRKRISN	0.217-0.564/ 0.459	RBD
RAD-SCoV-19	347-368	FASVYAWNRKRISNCVADYSV	0.246-0580/ 0.474	RBD
RAD-SCoV-20	354-375	NRKRISNCVADYSVLYNSASF	0.235-0.586/ 0.452	RBD
RAD-SCoV-21	361-382	CVADYSVLYNSASFSTFKCYG	0.228-0.537/ 0.440	RBD
RAD-SCoV-22	368-389	LYNSASFSTFKCYGVSPTKLN	0.250-0.542/ 0.462	RBD
RAD-SCoV-23	375-396	STFKCYGVSPTKLNDLCFTNV	0.247-0.570/ 0.465	RBD
RAD-SCoV-24	382-403	VSPTKLNDLCFTNVYADSFVI	0.253-0.498/ 0.425	RBD
RAD-SCoV-25	389-410	DLCFTNVYADSF**VIRGDE**VRQ	0.234-0.561/ 0.452	RBD
RAD-SCoV-26	396-417	YADSFVIRGDEVRQIAPGQTG	0.238-0.578/	RBD

			0.478	
RAD-SCoV-27	403-424	RGDEVRQIAPGQTGKIADYNY	0.260-0.591/ 0.492	RBD
RAD-SCoV-28	410-431	IAPGQTGKIADYNYKLPDDFT	0.238-0.582/ 0.479	RBD
RAD-SCoV-29	417-438	KIADY**NYKLP**DDFTGCVIAWN	0.236-0.566/ 0.429	RBD
RAD-SCoV-30	424-445	KLPDDFTGCVIAWN**SNNL**DSK	0.262-0.545/ 0.418	RBD
RAD-SCoV-31	431-452	GCVIAWNSNNLDSKVGGNYNY	0.231-0.640/ 0.503	RBD
RAD-SCoV-32	438-459	SNNLDSKVGGNYNYLYRLFRK	0.268-0.597/ 0.484	RBD
RAD-SCoV-33	445-466	VGGNYNYLYRLFRKSNLKPFE	0.245-0.528/ 0.438	RBD
RAD-SCoV-34	452-473	LYRLFRKSNLKPFERDISTEI	0.266-0.578/ 0.499	RBD
RAD-SCoV-35	459-480	SNLKPFERDI**STEIYQ**AGSTP	0.250-0.530/ 0.450	RBD
RAD-SCoV-36	466-487	RDISTEIYQAGSTPCNGVEGF	0.259-0.593/ 0.488	RBD
RAD-SCoV-37	473-494	YQAGSTPCNGVEGFNCYFPLQ	0.257-0.558/ 0.467	RBD
RAD-SCoV-38	480-501	CNGVEGFNCYFP**LQSYG**FQPT	0.246-0.524/ 0.440	RBD
RAD-SCoV-39	487-508	NCYFPLQSYGFQPTNGVGYQP	0.228-0.582/ 0.475	RBD
RAD-SCoV-40	494-515	SYGFQPTNGVGYQPYRVVVLS	0.251-0.597/ 0.476	RBD
RAD-SCoV-41	501-522	NGVGYQPYRVVVLSFELLHAP	0.238-0.484/ 0.417	RBD
RAD-SCoV-42	508-529	YRVVVLS**FELLHAPA**TVCGPK	0.240-0.509/ 0.437	RBD
RAD-SCoV-43	515-536	FELLHAPATVCGPKKSTNLVK	0.261-0.612/ 0.483	RBD
RAD-SCoV-44	452-494	LYRLFRKSNLKPFERDISTEIYQAGSTPCNGVEG FNCYFPLQ	0.268-0.590/ 0.517	RBD
RAD-Scaffold	RAD Control	-	-/-	

Table S2. Values of the absorbance from the ELISAs performed with the anti-RAD-SCoV-epitopes produced in mouse c57bl/6. Antibodies were tested against the RAD-scaffold, RAD-SCoV-epitopes and S-RBD purified proteins. Samples were measured from serum dilution of 1:160. n/a = not applicable as epitope outside RBD.

Antibody	Epitope Position	RAD-SCoV-epitope	RAD-Scaffold	RAD-SCoV-epitope minus RAD-Scaffold	S-RBD
Anti_RAD-SCoV-17	RBD	1.434	0.915	0.519	0.503
Anti_RAD-SCoV-11	S1	1.351	0.788	0.563	n/a
Anti_RAD-SCoV-38	RBD-ACE2	1.255	0.898	0.357	0.217

Anti_RAD-SCoV-39	RBD-ACE2	1.25	1.046	0.204	0.195
Anti_RAD-SCoV-42	RBD	1.249	1.079	0.17	0.346
Anti_RAD-SCoV-30	RBD	1.148	0.95	0.198	0.236
Anti_RAD-SCoV-18	RBD	1.079	0.822	0.257	0.442
Anti_RAD-SCoV-29	RBD	0.973	0.452	0.521	0.236
Anti_RAD-SCoV-37	RBD-ACE2	0.969	0.329	0.64	0.274
Anti_RAD-SCoV-44	RBD	0.839	0.214	0.625	0.200
Anti_RAD-SCoV-25	RBD	0.697	0.562	0.135	0.239
Anti_RAD-SCoV-22	RBD	0.662	0.123	0.539	0.264
Anti_RAD-SCoV-07	S1	0.647	0.19	0.457	n/a
Anti_RAD-SCoV-31	RBD	0.418	0.207	0.211	0.419

Table S3. List of oligonucleotides used in this work.

RAD-SCoV_01	F	GGCGGCGGGCTTAAGTGTGTGAATTTAACAACCCGGACTCAGTTACCCCCTGCATACACTAATAGCTTCACTAGAC
	R	CCGCCTCCCTTAAGTCTAGTGAAGCTATTAGTGTATGCAGGGGGTAACTGAGTCCGGGTTGTTAAATTCACACAC
RAD-SCoV_02	F	GGCGGCGGGCTTAAGTTTTGCAATGACCCGTTCTTGGGCGTCTACTACCATAAGAATAATAAGTCCTGGATGGAGC
	R	CCGCCTCCCTTAAGCTCCATCCAGGACTTATTATTCTTATGGTAGTAGACGCCCAAGAACGGGTCATTGCAAAAC
RAD-SCoV_03	F	GGCGGCGGGCTTAAGCATACCCCAATTAACCTGGTTCGGGATCTGCCACAAGGCTTCTCAC
	R	CCGCCTCCCTTAAGTGAGAAGCCTTGTGGCAGATCCCGAACCAGGTTAATTGGGGTATGC
RAD-SCoV_04	F	GGCGGCGGGCTTAAGCACACTCCTATCAACTTAGTGCGTGATCTTCCACAACACAAAGTTACGTTAAGATCCTACTTAACCCCTGGCGACTCATCGTCCGGCTGGACAGCCGGTGCTGCCC
	R	CCGCCTCCCTTAAGGGCAGCACCGGCTGTCCAGCCGGACGATGAGTCGCCAGGGGTTAAGTAGGATCTTAACGTAACTTTGTGTTGTGGAAGATCACGCACTAAGTTGATAGGAGTGTGC
RAD-SCoV_05	F	GGCGGCGGGCTTAAGGAGAATGGTACAATCACGGATGCAGTGGATTGTGCTC
	R	CCGCCTCCCTTAAGAGCACAATCCACTGCATCCGTGATTGTACCATTCTCC
RAD-SCoV_06	F	GGCGGCGGGCTTAAGGGGGATGAGGTACGCCAAATTGCGCCAGGCCAAACGGGCAAGATCGCGGACTATAACTACAAATTACCCGATGATC
	R	CCGCCTCCCTTAAGATCATCGGGTAATTTGTAGTTATAGTCCGCGATCTTGCCCGTTTGGCCTGGCGCAATTTGGCGTACCTCATCCCCC
RAD-SCoV_07	F	GGCGGCGGGCTTAAGAGTAATAACCTGGACTCCAAGGTCGGTGGAAATC
	R	CCGCCTCCCTTAAGATTTCCACCGACCTTGGAGTCCAGGTTATTACTC
RAD-SCoV_08	F	GGCGGCGGGCTTAAGTTAAAGCCTTTTGAACGTGACATCTCCACCGAAATCTACCAGGCTGGTAGCACGCCATGTAATGGTC
	R	CCGCCTCCCTTAAGACCATTACATGGCGTGCTACCAGCCTGGTAGATTTCGGTGGAGATGTCACGTTCAAAAGGCTTTAAC

RAD-SCoV_09	F	GGCGGCGGGCTTAAGGCGACAGTTTGCGGTCCCAAAAAATCGACAAATC
	R	CCGCCTCCCTTAAGATTTGTCGATTTTTGGGACCGCAAACTGTCGCC
RAD-SCoV_10	F	GGCGGCGGGCTTAAGTTTGGCCGTGATATTGCCGATACCACCGACGCTGTCCGCGA CCCGCAAACCTTAGAAATCC
	R	CCGCCTCCCTTAAGGATTTCTAAGGTTTGCGGGTCGCGGACAGCGTCGGTGGTATC GGCAATATCACGGCCAAAC
RAD-SCoV_11	F	GGCGGCGGGCTTAAGCACGCCGACCAGTTGACACCGACTTGGCGCGTGTATTCTAC AGGATCAAATGTCTTTCAGAC
	R	CCGCCTCCCTTAAGTCTGAAAGACATTTGATCCTGTAGAATACACGCGCCAAGTCG GTGTCAACTGGTCGGCGTGC
RAD-SCoV_12	F	GGCGGCGGGCTTAAGCGATTGGGGCCGGCATCTGTGCTAGTTATCAAACGCAAACT AATTCCCCTCGTCGTGCACC
	R	CCGCCTCCCTTAAGGTGCACGACGAGGGGAATTAGTTTGCGTTTGATAACTAGCAC AGATGCCGGCCCCAATCGC
RAD-SCoV_13	F	GGCGGCGGGCTTAAGTGAAACAAATCTATAAGACGCCACCCATTAAGGATTTTGGA GGGTTTAATTTCTCTCAAAC
	R	CCGCCTCCCTTAAGTTTGAGAGAAATTAAACCCTCCAAAATCCTTAATGGGTGGCG TCTTATAGATTTGTTTCAC
RAD-SCoV_14	F	GGCGGCGGGCTTAAGTCCTGCCCGATCCATCCAAGCCGTCCAAGCGTTCCTTTATTG AGGACCTTTTATTTAACAC
	R	CCGCCTCCCTTAAGTGTTAAATAAAAGGTCCTCAATAAAGGAACGCTTGGACGGCT TGGATGGATCGGGCAGGAC
RAD-SCoV_15	F	GGCGGCGGGCTTAAGGTCTACGATCCATTGCAGCCAGAACTGGACTCATTCC
	R	CCGCCTCCCTTAAGGAATGAGTCCAGTTCTGGCTGCAATGGATCGTAGACC
RAD-SCoV_16	F	GGCGGCGGGCTTAAGGACAGCTTGTCCAGTACGC
	R	CCGCCTCCCTTAAGCGTACTGGACAAGCTGTCC
RAD-SCoV-17	F1	GGCGGCGGGCTTAAGACTAACCTGTGCCCGTTCGGTGAAGTTTTCAACGC
	F2	GACCCGTTTCGCGTCCGTCTACGCGTGGC
	R1	CCGCCTCCCTTAAGCCACGCGTAGACGGACGCGAAACGGGTCGCGTTGAA
	R2	AACTTCACCGAACGGGCACAGGTTAGTC
RAD-SCoV-18	F1	GGCGGCGGGCTTAAGGAAGTTTTCAACGCGACCCGTTTCGCGTCCGTCTA
	F2	CGCGTGGAACCGTAAACGTATCTCTAACC
	R1	CCGCCTCCCTTAAGGTTAGAGATACGTTTACGGTTCCACGCGTAGACGGA
	R2	CGCGAAACGGGTCGCGTTGAAAACTTCC
RAD-SCoV-19	F1	GGCGGCGGGCTTAAGTTCGCGTCCGTCTACGCGTGGAACCGTAAACGTAT
	F2	CTCTAACTGCGTTGCGGACTACTCTGTCC
	R1	CCGCCTCCCTTAAGGACAGAGTAGTCCGCAACGCAGTTAGAGATACGTTT
	R2	ACGGTTCCACGCGTAGACGGACGCGAAC
RAD-SCoV-20	F1	GGCGGCGGGCTTAAGAACCGTAAACGTATCTCTAACTGCGTTGCGGACTA
	F2	CTCTGTCCTGTACAACTCTGCGTCTTTCC
	R1	CCGCCTCCCTTAAGGAAAGACGCAGAGTTGTACAGGACAGAGTAGTCCGC
	R2	AACGCAGTTAGAGATACGTTTACGGTTC
RAD-SCoV-21	F1	GGCGGCGGGCTTAAGTGCGTTGCGGACTACTCTGTCCTGTACAACTCTGC
	F2	GTCTTTCTCTACCTTTAAATGCTACGGTC
	R1	CCGCCTCCCTTAAGACCGTAGCATTTAAAGGTAGAGAAAGACGCAGAGTT
	R2	GTACAGGACAGAGTAGTCCGCAACGCAC
RAD-SCoV-22	F1	GGCGGCGGGCTTAAGCTGTACAACTCTGCGTCTTTCTCTACCTTTAAATG
	F2	CTACGGTGTTTCTCCGACCAAACTGAACC
	R1	CCGCCTCCCTTAAGGTTCAGTTTGGTCGGAGAAACACCGTAGCATTTAAA
	R2	GGTAGAGAAAGACGCAGAGTTGTACAGC
RAD-SCoV-23	F1	GGCGGCGGGCTTAAGTCTACCTTTAAATGCTACGGTGTTTCTCCGACCAA

	F2	ACTGAACGACCTCTGCTTTACCAACGTTC
	R1	CCGCCTCCCTTAAGAACGTTGGTAAAGCAGAGGTCGTTCAGTTTGGTCGG
	R2	AGAAACACCGTAGCATTTAAAGGTAGAC
RAD-SCoV-24	F1	GGCGGCGGGCTTAAGGTTTCTCCGACCAAACTGAACGACCTCTGCTTTAC
	F2	CAACGTTTATGCCGACTCTTTCGTTATCC
	R1	CCGCCTCCCTTAAGGATAACGAAAGAGTCGGCATAAACGTTGGTAAAGCA
	R2	GAGGTCGTTCAGTTTGGTCGGAGAAACC
RAD-SCoV-25	F1	GGCGGCGGGCTTAAGGACCTCTGCTTTACCAACGTTTATGCCGACTCTTT
	F2	CGTTATCCGTGGTGACGAAGTTCGTCAGC
	R1	CCGCCTCCCTTAAGCTGACGAACTTCGTCACCACGGATAACGAAAGAGTC
	R2	GGCATAAACGTTGGTAAAGCAGAGGTCC
RAD-SCoV-26	F1	GGCGGCGGGCTTAAGTATGCCGACTCTTTCGTTATCCGTGGTGACGAAGT
	F2	TCGTCAGATCGCGCCAGGTCAAACCGGCC
	R1	CCGCCTCCCTTAAGGCCGGTTTGACCTGGCGCGATCTGACGAACTTCGTC
	R2	ACCACGGATAACGAAAGAGTCGGCATAC
RAD-SCoV-27	F1	GGCGGCGGGCTTAAGCGTGGTGACGAAGTTCGTCAGATCGCGCCAGGTCA
	F2	AACCGGCAAAATCGCGGATTACAATTACC
	R1	CCGCCTCCCTTAAGGTAATTGTAATCCGCGATTTTGCCGGTTTGACCTGG
	R2	CGCGATCTGACGAACTTCGTCACCACGC
RAD-SCoV-28	F1	GGCGGCGGGCTTAAGATCGCGCCAGGTCAAACCGGCAAAATCGCGGATTA
	F2	CAATTACAAACTGCCGGACGATTTCACCC
	R1	CCGCCTCCCTTAAGGGTGAAATCGTCCGGCAGTTTGTAATTGTAATCCGC
	R2	GATTTTGCCGGTTTGACCTGGCGCGATC
RAD-SCoV-29	F1	GGCGGCGGGCTTAAGAAAATCGCGGATTACAATTACAAACTGCCGGACGA
	F2	TTTCACCGGTTGTGTTATTGCTTGGAACC
	R1	CCGCCTCCCTTAAGGTTCCAAGCAATAACACAACCGGTGAAATCGTCCGG
	R2	CAGTTTGTAATTGTAATCCGCGATTTTC
RAD-SCoV-30	F1	GGCGGCGGGCTTAAGAAACTGCCGGACGATTTCACCGGTTGTGTTATTGC
	F2	TTGGAACTCTAACAACCTGGACTCTAAAC
	R1	CCGCCTCCCTTAAGTTTAGAGTCCAGGTTGTTAGAGTTCCAAGCAATAAC
	R2	ACAACCGGTGAAATCGTCCGGCAGTTTC
RAD-SCoV-31	F1	GGCGGCGGGCTTAAGGGTTGTGTTATTGCTTGGAACTCTAACAACCTGGA
	F2	CTCTAAAGTTGGTGGTAACTACAACTACC
	R1	CCGCCTCCCTTAAGGTAGTTGTAGTTACCACCAACTTTAGAGTCCAGGTT
	R2	GTTAGAGTTCCAAGCAATAACACAACCC
RAD-SCoV-32	F1	GGCGGCGGGCTTAAGTCTAACAACCTGGACTCTAAAGTTGGTGGTAACTA
	F2	CAACTACCTGTATCGTCTGTTCCGTAAAC
	R1	CCGCCTCCCTTAAGTTTACGGAACAGACGATACAGGTAGTTGTAGTTACC
	R2	ACCAACTTTAGAGTCCAGGTTGTTAGAC
RAD-SCoV-33	F1	GGCGGCGGGCTTAAGGTTGGTGGTAACTACAACTACCTGTATCGTCTGTT
	F2	CCGTAAATCTAACCTCAAACCATTTGAAC
	R1	CCGCCTCCCTTAAGTTCAAATGGTTTGAGGTTAGATTTACGGAACAGACG
	R2	ATACAGGTAGTTGTAGTTACCACCAACC
RAD-SCoV-34	F1	GGCGGCGGGCTTAAGCTGTATCGTCTGTTCCGTAAATCTAACCTCAAACC
	F2	ATTTGAACGTGACATCAGCACCGAAATCC

	R1	CCGCCTCCCTTAAGGATTTCGGTGCTGATGTCACGTTCAAATGGTTTGAG
	R2	GTTAGATTTACGGAACAGACGATACAGC
RAD-SCoV-35	F1	GGCGGCGGGCTTAAGTCTAACCTCAAACCATTTGAACGTGACATCAGCAC
	F2	CGAAATCTACCAGGCGGGTTCTACCCCGC
	R1	CCGCCTCCCTTAAGCGGGGTAGAACCCGCCTGGTAGATTTCGGTGCTGAT
	R2	GTCACGTTCAAATGGTTTGAGGTTAGAC
RAD-SCoV-36	F1	GGCGGCGGGCTTAAGCGTGACATCAGCACCGAAATCTACCAGGCGGGTTC
	F2	TACCCCGTGCAACGGTGTCGAGGGTTTCC
	R1	CCGCCTCCCTTAAGGAAACCCTCGACACCGTTGCACGGGGTAGAACCCGC
	R2	CTGGTAGATTTCGGTGCTGATGTCACGC
RAD-SCoV-37	F1	GGCGGCGGGCTTAAGTACCAGGCGGGTTCTACCCCGTGCAACGGTGTCGA
	F2	GGGTTTCAACTGCTACTTCCCGCTGCAGC
	R1	CCGCCTCCCTTAAGCTGCAGCGGGAAGTAGCAGTTGAAACCCTCGACACC
	R2	GTTGCACGGGGTAGAACCCGCCTGGTAC
RAD-SCoV-38	F1	GGCGGCGGGCTTAAGTGCAACGGTGTCGAGGGTTTCAACTGCTACTTCCC
	F2	GCTGCAGTCTTATGGTTTCCAGCCTACTC
	R1	CCGCCTCCCTTAAGAGTAGGCTGGAAACCATAAGACTGCAGCGGGAAGTA
	R2	GCAGTTGAAACCCTCGACACCGTTGCAC
RAD-SCoV-39	F1	GGCGGCGGGCTTAAGAACTGCTACTTCCCGCTGCAGTCTTATGGTTTCCA
	F2	GCCTACTAACGGCGTTGGTTACCAGCCGC
	R1	CCGCCTCCCTTAAGCGGCTGGTAACCAACGCCGTTAGTAGGCTGGAAACC
	R2	ATAAGACTGCAGCGGGAAGTAGCAGTTC
RAD-SCoV-40	F1	GGCGGCGGGCTTAAGTCTTATGGTTTCCAGCCTACTAACGGCGTTGGTTA
	F2	CCAGCCGTACCGTGTTGTTGTTCTGTCTC
	R1	CCGCCTCCCTTAAGAGACAGAACAACAACACGGTACGGCTGGTAACCAAC
	R2	GCCGTTAGTAGGCTGGAAACCATAAGAC
RAD-SCoV-41	F1	GGCGGCGGGCTTAAGAACGGCGTTGGTTACCAGCCGTACCGTGTTGTTGT
	F2	TCTGTCTTTCGAACTGCTGCACGCTCCTC
	R1	CCGCCTCCCTTAAGAGGAGCGTGCAGCAGTTCGAAAGACAGAACAACAAC
	R2	ACGGTACGGCTGGTAACCAACGCCGTTC
RAD-SCoV-42	F1	GGCGGCGGGCTTAAGTACCGTGTTGTTGTTCTGTCTTTCGAACTGCTGCA
	F2	CGCTCCTGCTACCGTTTGTGGCCCGAAAC
	R1	CCGCCTCCCTTAAGTTTCGGGCCACAAACGGTAGCAGGAGCGTGCAGCAG
	R2	TTCGAAAGACAGAACAACAACACGGTAC
RAD-SCoV-43	F1	GGCGGCGGGCTTAAGTTCGAACTGCTGCACGCTCCTGCTACCGTTTGTGG
	F2	CCCGAAAAAAAGCACCAATCTGGTTAAAC
	R1	CCGCCTCCCTTAAGTTTAACCAGATTGGTGCTTTTTTTCGGGCCACAAAC
	R2	GGTAGCAGGAGCGTGCAGCAGTTCGAAC

Figures

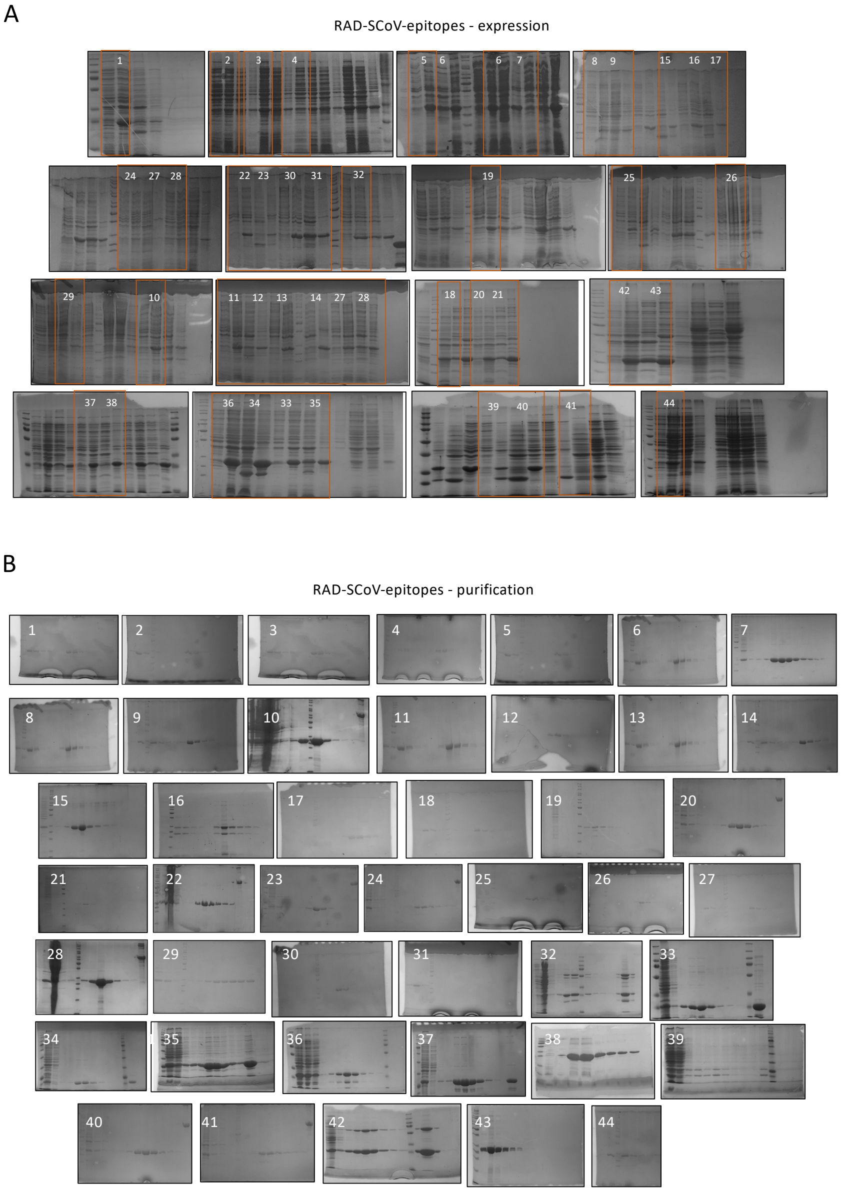

Figure S1. Production of the recombinant RAD-SCoV-epitopes. (**A**) Recombinant proteins were expressed in *E. coli* BL21(DE3) cells carrying pUB25S and the pRAD-SCoV-peptides vectors after induction with 0.4 mM IPTG for 6 h at 25°C. SDS-PAGE for each construct showing the non-induced and induced bacterial extracts. (**B**) The proteins were purified by IMAC and polished with a SEC shown in the SDS-PAGE.

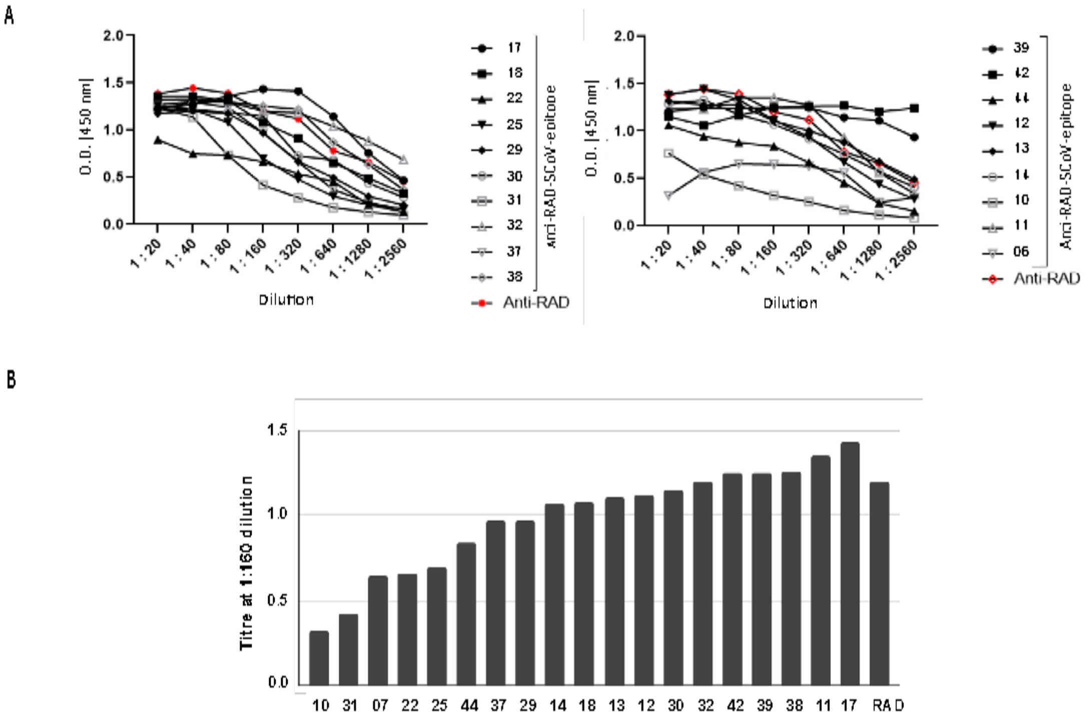

Figure. S2 Evaluation of the immunogenic profile of the RAD-SCoV-epitopes after immunisation. ELISA was performed using the sera antibodies generated in mouse C57BL/6 against the RAD-SCoV-epitopes (**A**). The graphs show the titres of antibodies generated after the third immunisation. Each serum was tested against the corresponding antigen. (**B**) Comparison of the antibodies titre in the dilution of 1:160.

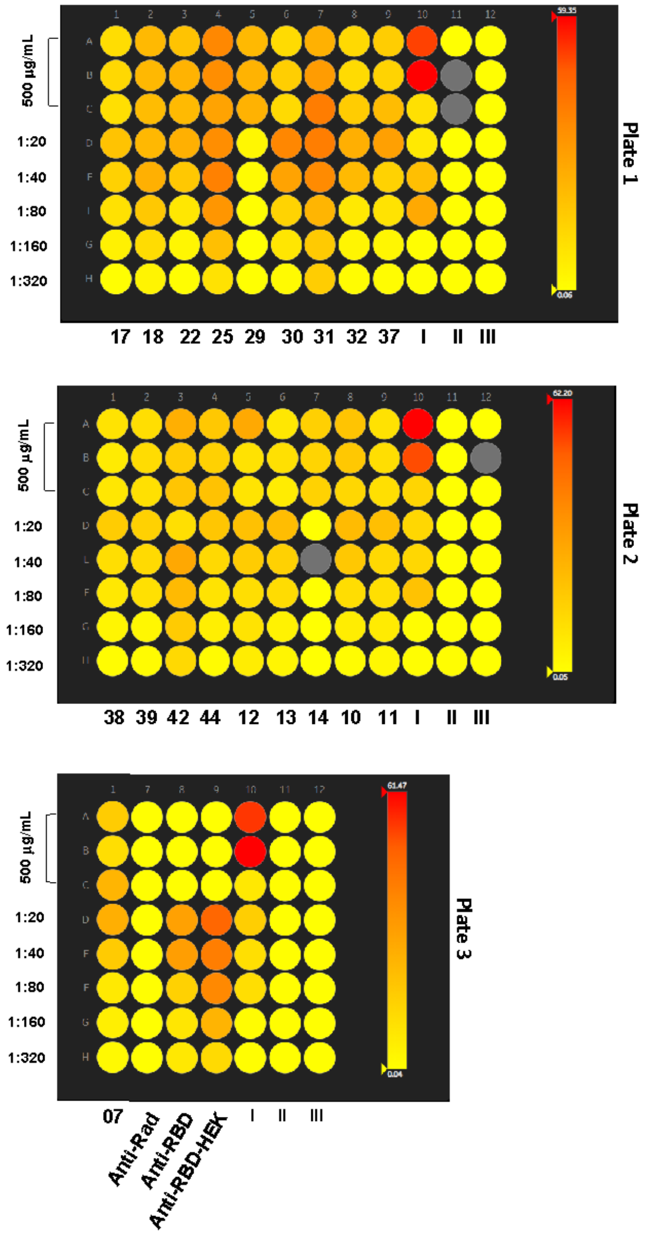

Figure S3. Heatmap of all the plates used in the immunofluorescence analysis of VERO CCL-81 cells infected in the presence of SARS-CoV-2 and the antibodies against anti-SCoV-RAD-epitopes. (I) A10 and B10: control convalescent serum (primary) and Alexa-488 (secondary) infected cells; C10 and D10: control anti-RBD produced in mouse (primary) and Alexa-488 (secondary) infected cells; E10 and F10: control anti-RBD produced in mouse (primary) and Alexa-488 (secondary) non-infected cells; G10 and H10: control anti-RBD produced in mouse (primary) and Alexa-488 (secondary) non-infected cells. (II) A11 and B11:

only Alexa-488 anti-human IgG (secondary) infected cells; C11 and D11: only Alexa-488 anti-human IgG (secondary) infected cells; E11 and F11: only Alexa-488 anti-human IgG (secondary) non-infected cells; G11 and H11: only Alexa-488 anti-human IgG (secondary) non-infected cells. (III) A12, B12, C12 and D12: only infected cells; E12, F12, G12 and H12: only non-infected cells.

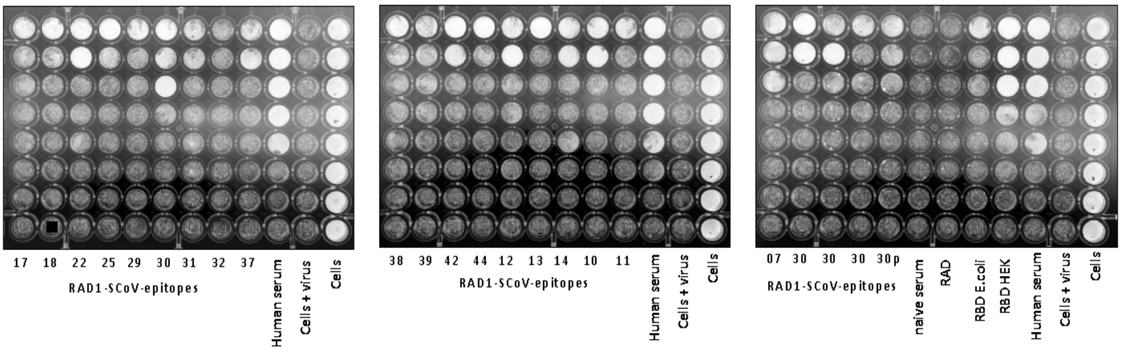

Figure S4. Plates from the neutralisation assays with antibodies anti-RAD-SCoV-epitopes produced in mice. Original images of the 96-well plates in virus neutralisation assays which were used to generate the composite image in Fig. 5.
